# Clinico - Radiological Results of Tibial Bicondylar Fractures Managed with Ilizarov Technique with or without Minimal Internal Fixation

**DOI:** 10.5704/MOJ.2203.004

**Published:** 2022-03

**Authors:** AA Kawoosa, SA Mantoo, N Ali, GN Dar

**Affiliations:** Department of Orthopaedics, Government Medical College Srinagar, Jammu and Kashmir, India

**Keywords:** Ilizarov, tibial plateau fractures, Schatzker V and VI, tibial bicondylar fractures

## Abstract

**Introduction:**

Tibial bicondylar fractures are difficult fractures to treat and are usually associated with complications.

**Materials and methods:**

Thirty-five patients with Schatzker type V and VI fractures were managed from June 2016 to July 2018 with Ilizarov technique. The mean age of the patients was 46.5 ± 8.9 years, with 28 male and seven female patients. Sixteen patients had Schatzker type V fracture and the remaining had type VI. The functional outcome was assessed by using Modified functional evaluation system by Karlstrom - Olerud and the radiological outcome by Rasmussen's Radiological Score (RRS).

**Results:**

All patients achieved radiological union at a mean duration of 16 weeks for type 5 and 17 weeks for type 6 however, full weight-bearing was allowed at a mean of 18 weeks (14 - 22 weeks). Functional results were excellent in 24 cases, good in 10 and poor in one. Most patients achieved functional range of motion at the knee joint (average flexion 1280) except one, who had a flexion of less than 1100. One patient with a delayed union united after bone marrow injection. Other complications included pin tract infections in 9 cases, axial malalignment of less than 10^0^ in 4 cases and a prominent screw in one.

**Conclusion:**

Percutaneous restoration of articular anatomy and a ring external fixation with or without minimal internal fixation is an excellent method of treatment in this group of fractures caused by high energy trauma and with a usual association of severe comminution and a poor soft tissue envelope.

## Introduction

Articular fractures of the proximal tibia also known as tibial plateau or tibial condylar fractures involve a major weight bearing joint and are serious injuries that frequently result in functional impairment. These fractures are caused by considerable high energy trauma, not only causing severe bony comminution but also considerable soft tissue insults. Owing to the presence of articular depression, severe comminution and associated soft tissue and ligamentous injuries these fractures are difficult to manage. The usual association of neurovascular compromise and compartment syndrome make management even more challenging. Failure to restore articular congruity, presence of ligamentous instability and knee stiffness are the most important factors leading to poor outcome. Non-operative management yields poor results and the mainstay of treatment in patients with Schatzker V and VI fractures has been open reduction and internal fixation. However open reduction and internal fixation is invariably complicated by the condition of the soft tissue envelope of the proximal tibia, that may lead to wound break-down, skin necrosis, deep infections, and stiffness depending on the extent of the injury^1,2,3^. High incidence of infection has been reported with dual plating through two incisions and the incidence of deep infection being even higher in cases of open fractures^4^. An infection rate of 2.6% to 45% has been reported in a recent systematic review that require several revisions often leading to unsatisfactory outcome^5^. Joint stiffness and mal-union are other common complications^6^. In order to avoid these complications, methods with minimal invasion and limited internal fixation have been attempted with good results.

Ilizarov external fixation is one such attractive treatment option to address all treatment goals. The merits of Ilizarov are closed or mini-open fracture reduction with less chance of wound complications, early joint motion, functional

loading and weight bearing, possibility of post-operative fine tuning of alignment and faster return to function. Even in cases of severe comminution that might ultimately require replacement, this methodology allows an easier conversion due to absence of large incisions, soft tissue compromise and the absence of hardware inside.

A well-planned technique based on the understanding of fracture pattern on CT scan, minimal invasion to restore the articular congruity, minimal internal fixation wherever needed and a ring fixation using counteracting olives for compression appears to be an effective method of treatment. At our institution we have been routinely treating these difficult fractures with this methodology. This prospective study aimed at objective assessment of clinico -radiological results of this technique.

## Materials and Methods

**Fig. 1:**
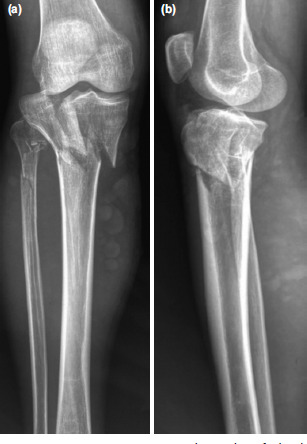
Case No.12, pre-operative radiographs of the knee showing Shatzker Type VI fracture of the proximal tibia (a). Anteroposterior view. (b) Lateral view.

**Fig. 2:**
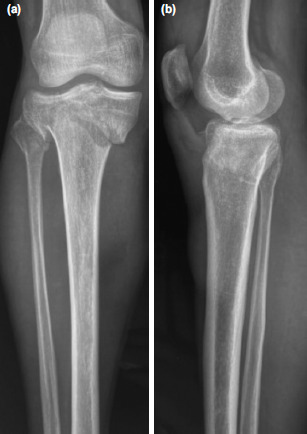
Case No.7, pre-operative radiographs of the knee showing Shatzker Type VI fracture. (a) Antero-posterior view. (b) Lateral view.

**Fig. 3:**
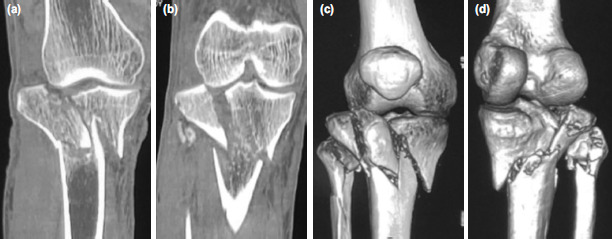
Case No.12, pre-operative CT images. (a) Coronal section image showing Shatzker Type VI fracture. (b) Coronal section image showing tibial condylar widening and intra-articular step. (c) 3D reconstruction view anterior aspect. (d) 3D reconstruction view posterior aspect.

**Fig. 4:**
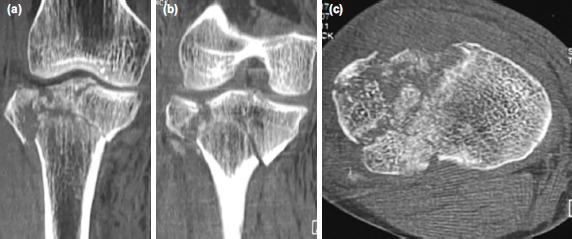
Case No.7, CT images of the knee showing comminution of the proximal tibia. (a) Coronal section image of anterior part of the tibial condyle. (b) Coronal section image of posterior part of the tibial condyle. (c) Cross-section of the tibial plateau revealing comminution.

Between June 2016 and July 2018, 35 patients with type V and VI tibial plateau fractures were managed with Ilizarov circular external fixator. All the patients in the age group of 18-70 years, with an open (except open type IIIC fractures) or closed Schatzker type V and VI fractures were included in this study. This prospective study was approved by the Institutional ethical committee. After routine radiography, (Fig. 1 and Fig. 2) computed tomography was performed in all cases to assess the degree of comminution, the amount of articular depression, to identify the main fragments that were amenable to a limited internal fixation (screws) and to understand the fracture geometry to plan different counteracting olive wires for secure fixation (Fig. 3 and Fig. 4). The open fractures were classified according to GustiloAnderson classification system and soft-tissue injuries associated with closed fractures were classified according to the Tscherne classification system.

**Fig. 5:**
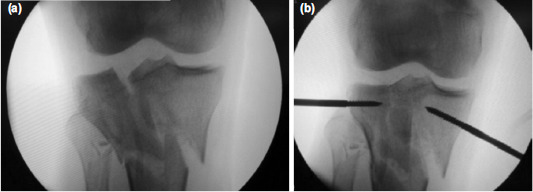
Case No.12, intra-operative fluoroscopic images. (a) Before reduction, (b) after closed reduction of the medial and lateral condylar fragments using joystick technique.

**Fig. 6:**
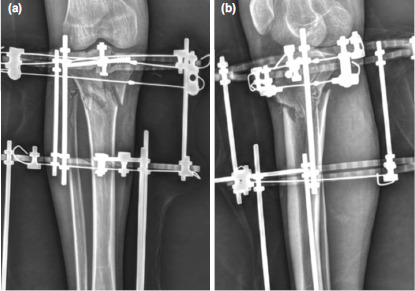
Case No.12, post-operative radiographs of the knee. (a) Antero-posterior view showing restoration of the condylar width, reduction of intra-articular step and stabilisation with an inter-fragmentary screw and counter acting olive wires using a ring fixator. (b) Lateral view showing well maintained posterior tibial slope.

**Fig. 7:**
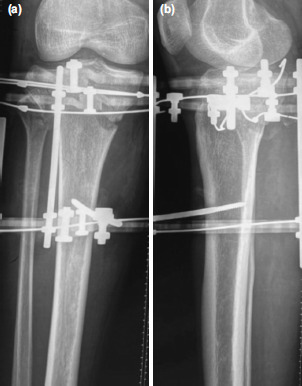
Case No.7, post-operative radiographs. (a) Anteroposterior view showing olive wore fixation through fibular head for buttressing of latera condyle. (b) Lateral view.

**Fig. 8:**
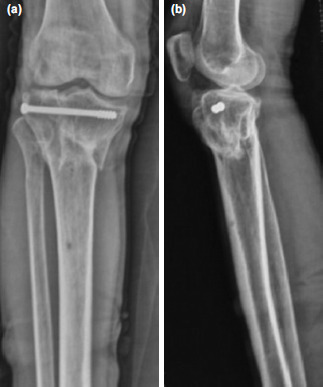
Case No.12, radiographs at final follow up showing union. (a) Antero-posterior view. (b) Lateral view.

**Fig. 9:**
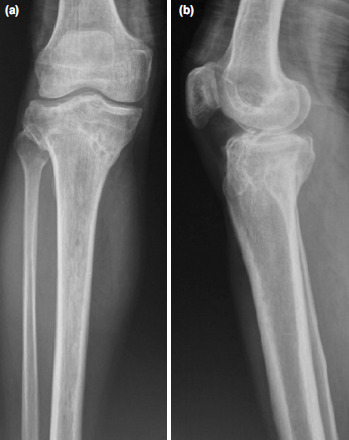
Case No.7, radiographs of knee at final follow up showing fracture union and well-maintained alignment. (a) Antero-posterior view. (b) Lateral view.

The surgery was performed without tourniquet on a traction table and the axial reduction was achieved by traction. The joint surface was reconstructed wherever necessary, using closed techniques like manual pressure, reduction clamps or percutaneous elevation using elevators, joy sticking with Steinmann pin, Schanz pin, thick K-wires or by olive wires (Fig 5). In cases with central depression a small window was made to aid in reconstruction of the articular surface. Once the articular surface was satisfactorily restored the ring fixation was performed as per the pre-operative planning for olive wire placement (Fig. 6 and Fig. 7). Fractures with large condylar fragments were fixed with cannulated screws to achieve inter-fragmentary compression prior to ring fixation. A three-ring construct was used for fixation with the proximal ring at the level of fibular head and the distal one just above ankle with a middle ring close to the fracture site. Fixation was performed as per the pre-operative plan and as per the safety zones at the respective levels. Three and sometimes four olive wires, at least 15mm distal to the joint line with a total divergence of minimum 60°, were commonly required for stabilisation of the condylar and metaphyseal fragments. An olive wire through the head of fibula was used as a buttress for the lateral tibial condyle. As a hybrid modification half pins were used in the middle and distal rings to avoid large muscular areas. Metaphyseal bone defect if any was filled with bone graft through the bone window on the medial or lateral side of the proximal tibia. Pins were antiseptically dressed and any small wounds sutured and dressed. The post-operative radiographs were assessed for any persistent gap, angulation or translation left over at the metaphyseo-diaphyseal junction and was corrected in the immediate post-operative period. Patients were discharged on the second post-operative day after non weight bearing ambulation and after demonstration of range of motion exercises. Each patient after discharge was followed-up at 2nd, 4th, 8th week and then at monthly intervals till patient resumed normal routine activities and finally at one year. At each follow-up patient was examined for pin tract problems like pain, irritation, or any discharge. External ring fixator was removed when radiological union at articular level, and metaphyseo-diaphyseal level was thought strong enough to withstand weight without causing collapse or condylar tilt (Fig. 8 and Fig. 9). The “Kurgan protocol”^7^ was used for post-operative pin site dressings and the Checketts-Otterburns classification was used to describe the pin site infections^8^. The fracture was considered united when antero-posterior and lateral radiographs showed a bridging callus of three out of four cortices and/or the fracture was stable when stressed manually and the patients were able to walk without pain after the connecting rods had been disengaged. At this stage patients were allowed progressive weight bearing and the ring fixator was later removed without anaesthesia. After removal of fixator all patients were put in hinged knee ROM brace for additional four weeks. At the final follow-up of one year, quantification of the functional and clinical outcome was performed using modified functional evaluation according to KarlstromOlerud Scoring System. The system is based on eleven parameters with each having a maximum of three points and a minimum of 1 point (Table I). On the basis of the total score, the functional outcome was graded as excellent (33 points), good (30-32 points), satisfactory (27-29 points), moderate (24-26 points), and poor (21-23 points). Assessment of the bony status was performed using Modified Rasmussen Criteria which is based on the amount of articular depression, amount of condylar widening and degree of varus or valgus angulation (Table II). The radiological score was graded as excellent (9 points), good (7-8 points), fair (5-6 points) and poor (< 5 points).

**Table I:** Karlstrom Olerud Score evaluating the results in points

Parameter	3 points	2 points	1 point
Pain	No	Little	Severe
Difficulty in walking	No	Moderate	Severe/ Limp
Difficulty in stairs	No	Supported	Unable
Difficulty in previous sports	No	Some sports	Unable
Limitation at work	No	Moderate	Unable
Status of skin	Normal	Various colors	Ulcer/ fistula
Deformity	No	Little, up to 7o	Remarkable > 7o
Muscle atrophy	< 1 cm	1-2 cm	> cm
Leg length discrepancy	< 1 cm	1 - 2 cm	> 2 cm
Loss of motion at knee joint	< 10o	10 - 20o	> 20o
Loss of motion at subtalar joint	< 10o	10 - 20o	> 20o
**Grade**	**Points**		
Excellent	33		
Good	30 - 32		
Satisfactory	27 - 29		
Moderate	24 - 26		
Poor	21 - 23		

**Table II:** Modified Rasmussen criteria for radiological assessment

Radiological Parameters	Points
Articular depression	
None	3
≤ 5 mm	2
6-10 mm	1
> 10 mm	0
Condylar widening	
None	3
≤ 5 mm	2
6-10 mm	1
> 10 mm	0
Varus/ valgus angulation	
None	3
< 10°	2
10-20°	1
> 20°	0
**Grading**	**Points**
Excellent	9
Good	7-8
Fair	5-6
Poor	< 5

## Results

The patient demographics and fracture characteristics have been summarised (Table III). The mean interval between the injury and the surgical intervention was 7 days (range 3-11 days). In 18 patients the technique involved closed reduction and ligamentotaxis while as in 12 cases closed reduction, and an inter-fragmentary canulated screw fixation was used. Intra-operatively, site-specific elevation of the depressed articular fragments was performed in five cases to achieve articular congruity (Fig 5). Bone grafting to fill the large voids and defects was required in five patients and the graft was obtained from the ipsilateral iliac crest.

**Table III:** Patient demography and types of fracture

Details	n = No. of patients
Age (years)	46.5±8.9
Sex (M:F)	4:1
Side (R:L)	4:3
Schatzker type	
Type V	n = 16
Type VI	n = 19
Close or open injury	
Close	n = 32
Open (type 1)	n = 3
Soft tissue injury (Tscherne grading)	
Grade 0	n = 0
Grade I	n = 5
Grade II	n = 24
Grade III	n = 3

In 32 patients partial weight bearing was started between 1216 weeks and in 3 cases it was started after 16 weeks, with an average duration of 15 weeks. Full weight bearing in 31 cases was started between 16-20 weeks and in 4 cases full weight bearing was started after 20 weeks with an average duration of 18 weeks. Average time to radiological union was 16 weeks in type V and 17 weeks in type VI. In one patient, fracture took longer than 5 months to heal which was managed by bone marrow injection and ultimately united at 22 weeks.

Commonest complication of pin tract infection was seen in nine patients that however healed with regular dressing and oral antibiotics. In more than 90% of the patients, normal axial and coronal alignment was achieved as well as maintained in the ring. Besides, the posterior tibial slope was also corrected and maintained with an average of 8° at final follow-up. An axial mal-alignment of less than 10° varus in two patients and less than 10° valgus was seen in two patients however, no further intervention was performed in them. Varus mal-alignment developed secondary to weight bearing. Prominent screw, causing hardware symptoms in one patient, was removed after union under local anaesthesia. There were no instances of septic arthritis, no case of non-union, gross malunion or loss of reduction and none of the cases developed any distal neurovascular compromise following surgical management.

Eight patients had mild subjective complaints like occasional pain or subtle decrease in walking capacity. There was no direct correlation of such complaints with fracture type or radiographic score. Remaining 27 patients had no pain and had normal walking capacity but with varying radiological score. Most of the cases achieved full range of motion at the knee joint, with an average of 128° of flexion. Only one patient had a flexion of less than 110° and in 3 patients there was an extension lag of 5°. Three cases had some signs of instability on final follow-up however the degree of instability did not produce any subjective complaints and didn’t require any surgical treatment. Normal walking was observed in 33 patients while two patients walked with a mild limp. None of the patients used any walking aids. Squatting and stair climbing was normal in 32 patients while as three patients complained of mild limitation in squatting. Functional results were excellent in 24 cases, good in 10 and poor in one. The pre-operative articular depression was seen in 74% patients and ranged from 1mm to 9mm but on final follow-up articular depression was found in only 8 cases (23%) ranging from 1mm to 4mm only while in rest of the 27 cases there was no articular depression at the final followup. The mean functional score at one year follow-up was 32.11±1.95 and the mean radiological score was 8.37±0.96 and radiologically 22 patients were excellent (Score of 9), 12 were good (7-8) and one scored as poor (Score < 5).

A subgroup analysis of the results between Type V and Type VI fractures (Tables IV and V) revealed better results in the group with type V fractures. While the radiological scores in type V fractures improved from an average pre-operative score of 6 to an average post-operative score of 9, the same scores improved from an average pre-operative score of 6 to an average post-operative score of 8 in case of type VI fractures which is statistically significant (p – 1.26). Similarly the final functional results were seen better in type V (excellent 13 and 3 Good) as compared with the type VI fractures (Excellent 11 good 7 and one poor).

**Table IV:** Radiological and functional scores of patients with type V fractures

Pt.no	Modified Rasmussen radiological score based on articular depression, condylar widening and alignment		Karlstrom – Olerud grading
	Pre-operative	Post-operative	
08	07 (Good)	09 (Excellent)	Excellent
09	04 (Poor)	07 Good	Excellent
10	07 (Good)	07 (Good)	Excellent
11	04 (Poor)	09 (Excellent)	Excellent
12	05 (Fair)	09 (Excellent)	Excellent
15	04 (Poor)	09 (Excellent)	Excellent
17	08 (Good)	09 (Excellent)	Excellent
18	07 (Good)	09 (Excellent)	Excellent
19	06 (Fair)	09 (Excellent)	Excellent
21	07 (Good)	09 (Excellent)	Excellent
22	05 (Fair)	07 (Good)	Good
28	07 (Good)	08 (Good)	Good
29	08 (Good)	09 (Excellent)	Excellent
31	08 (Good)	09 (Excellent)	Excellent
32	05 (Fair)	09 (Excellent)	Excellent
34	06 (Fair)	09 (Excellent)	Good
	Average score = 06	Average score = 09	

**Table V:** Radiological and functional scores of patients with type VI fractures

Pt.no	Modified Rasmussen radiological score based on articular depression, condylar widening and alignment		Karlstrom – Olerud grading
	Pre-operative	Post-operative	
O1	06 (Fair)	08 (Good)	Excellent
02	07 (Good)	09 (Excellent)	Excellent
03	07 (Good)	09 (Excellent)	Excellent
04	04 (Poor)	07 (Good)	Good
05	05 (Fair)	09 (Excellent)	Excellent
06	06 (Fair)	09 (Excellent)	Excellent
O7	04 (Poor)	08 (Good)	Good
13	08 (Good)	09 (Excellent)	Excellent
14	06 (Fair)	08 (Good)	Excellent
16	08 (Good)	09 (Excellent)	Excellent
20	02 (Poor)	04 (Poor)	Poor
23	06 (Fair)	08 (Good)	Good
24	08 (Good)	09 (Excellent)	Good
25	07 (Good)	09 (Excellent)	Good
26	06 (Fair)	08 (Good)	Good
27	05 (Fair)	08 (Good)	Good
30	06 (Fair)	09 (Excellent)	Excellent
33	04 (Poor)	07 (Good)	Excellent
35	05 (Fair)	09 (Excellent)	Excellent
	Average pre-operative = 06	Average post-operative = 08	

## Discussion

Treatment of high-energy tibial plateau fractures like Schatzker type V and VI fractures remains difficult. Restoration of articular congruity is mandatory and careful treatment of soft tissues is as important as the bony injury itself. Open reduction and internal fixation have been the mainstay of treatment of these high energy fractures, the goal being anatomic restoration of the articular surface. However open reduction and internal fixation is associated with postoperative complications like infection, problems with wound healing and even at times wound dehiscence^2,4^. It is not uncommon to experience loss of reduction, collapse of articular fragments, malunion and non-unions even after open reduction and internal fixation^6^. The high rates of deep infection (18%) were reported by Canadian Orthopaedic Society^9^ while Shah *et al* reported an overall infection rate of 17% with deep infection being 13.8%. They attributed slightly lower deep infection rate to the waiting period till soft tissue healing^10^. Young *et al* in 1994 reported deep infection in seven of their eight patients that had been treated with plates^2^. Ruffulo *et al* recorded over all complication rate of 27.9% that included 23.6% deep infection and 10.0% nonunion and even higher rates of infection were found in Open fractures (43.8%) as compared to 21.0% for closed injuries^4^. A study in March 2020 reports infection rate of 20%^11^. Despite open reduction and internal fixation there are reports of malunion. Yu *et al* (2009) treated 65 cases of complex tibial plateau fractures by double buttress plating and reported knee stiffness in 9 cases, varus deformity in 3, valgus deformity in 2, wound infection in 2 (for which debridement of wound followed by external fixation were performed after implant removal), and post-traumatic osteoarthritis of knee in 10 cases for which 2 patients were operated for total knee arthroplasty at a duration of 1 year and 2 years post-operatively^6^. Another concern with open reduction and internal fixation is the loss of range of motion across knee, that is not well tolerated by the Asian population for their routine activities. Yao *et al* reported an average range of motion of 115° in a study on functional outcomes of bicondylar tibial plateau fractures treated with dual buttress plating^12^. Barei *et al* in their series reported accurate reduction in half of their cases and found significant residual dysfunction after dual plating for bicondylar fractures^13^.

During the past three decades, the development of new techniques and equipment has changed the approach in the treatment of these injuries towards minimally invasive surgery. The biological and mechanical advantages of the circular external fixator in the treatment of high velocity tibial plateau fractures is reflected by the results of present series with a 100% union rate and an excellent average range of motion across the knee. The preservation of full range of Knee motion in our population is of utmost importance for their daily activities like sitting cross legged and squatting. Using Ilizarov technique Raza *et al* reported an average range of motion of 120°^14^. Dendrinos *et al* using ilizarov technique in their series of 24 patients comprising of open fractures and complex injuries achieved union in all patients at an average of 14.4 weeks^15^. Similar approach of Ilizarov fixation with minimal internal fixation in selected cases reported satisfactory results^16^. Excellent to good results were reported by Catagni *et al* in their hybrid Ilizarov with minimal internal fixation^17^. Ferreira used limited open reduction, cannulated screw fixation and circular fixator in 46 cases of Schatzker V and VI tibial plateau fractures and achieved good results without any loos of reduction and wound complications^18^. A Multicenter, prospective, randomised clinical trial by Hall *et al* revealed that circular fixator group had lesser blood loss, spent less time in hospital and had superior early outcome in terms of HSS scores at six months while the number and severity of complications were higher with open reduction and internal fixation^19^. A recent metanalysis comparing Open reduction internal fixation with Ilizarov fixation concluded that the ring fixation had some advantages in terms of shorter hospital stay and faster return to pre-injury state^20^. While it is very easy to indirectly reduce, compress and hold large condylar fragments, the technique is particularly useful in dealing with the articular comminution by indirect means of ligamentotaxis. A temporary trans articular fixation for initial four to six weeks is being used in many centres to prevent early collapse in cases with severe comminution^16^. Except for some minor pin tract infections and few instances of malalignment in the present series there were no major complications as seen with open reduction and internal fixation. Slightly better outcome in cases of type V fractures than type VI fractures could be explained by the higher degree of comminution, depression and meta-diaphyseal dissociation in case of type 6 fractures that lead to more chances of malalignment and articular incongruence.

The absence of any major soft tissue complications, any significant malunion and radiologically evident restoration of articular surface in all of the patients strongly supports a view to consider Ilizarov technique of minimal invasion as the preferable treatment in these high velocity fractures. The less incidence of malunion in present series as well as other series using Ilizarov technique is due to the possibility of correcting any malalignment even in the post-operative period. The maximum preservation of soft issue envelope around the highly comminuted fracture fragments and the undisturbed fracture hematoma are responsible for the low incidence of non - union and deep infections with this technique. The successful outcome with this technique however is dependent on meticulous pre-operative understanding of fracture anatomy, intra-operative restoration of articular surface and stable fixation. While most of the articular congruence can be brought about by indirect means impacted fragments are dealt with fragment specific limited open reduction. The appropriate use of olive wires as per the pre-operative planning to bring about interfragmentary compression and modifications wherever deemed necessary like the use of interfragmentary screws to prevent future collapse are of paramount importance. The present study involves a statistically significant volume of patients with no loss to follow-up and includes objective clinical and radiological assessment of the results however it lacks a simultaneous comparative group treated with open reduction and internal fixation.

## Conclusion

Keeping in view the high complication rates with open reduction and internal fixation, management of the high energy tibial fractures with Ilizarov circular ring fixator with or with- out minimally invasive techniques appears to be an excellent method of treatment with minimal complications. This technique could be considered as the preferable treatment in such high velocity injuries with severe comminution of bony fragments and jeopardised soft tissue envelope. The disadvantages of bulky nature of the ring fixator could be minimised by hybrid modification in the form of 5/8th ring fixation in selected cases of stable condyles and an incorporation of uni-planner fixator distally.
